# Complete Genome Sequences of Escherichia coli Strains 1303 and ECC-1470 Isolated from Bovine Mastitis

**DOI:** 10.1128/genomeA.00182-15

**Published:** 2015-03-26

**Authors:** Andreas Leimbach, Anja Poehlein, Anika Witten, Flemming Scheutz, Ynte Schukken, Rolf Daniel, Ulrich Dobrindt

**Affiliations:** a Institute of Hygiene, University of Münster, Münster, Germany; b Department of Genomic and Applied Microbiology, Göttingen Genomics Laboratory, Institute of Microbiology and Genetics, Georg-August-University of Göttingen, Göttingen, Germany; c Institute for Molecular Infection Biology, Julius-Maximilians-University of Würzburg, Würzburg, Germany; d Institute for Human Genetics, University of Münster, Münster, Germany; e Statens Serum Institut, Copenhagen, Denmark; f Department of Population Medicine and Diagnostic Sciences, College of Veterinary Medicine, Cornell University, Ithaca, New York, USA; g GD Animal Health, Deventer, The Netherlands

## Abstract

Escherichia coli is the leading causative agent of acute bovine mastitis. Here, we report the complete genome sequence of E. coli O70:H32 strain 1303, isolated from an acute case of bovine mastitis, and E. coli Ont:Hnt strain ECC-1470, isolated from a persistent infection.

## GENOME ANNOUNCEMENT

The outcome and severity of E. coli intramammary infections were previously mainly associated with cow factors reacting to pathogen-associated molecular patterns rather than the genomic makeup of the infecting strain (1). Nevertheless, certain E. coli strains consistently cause an acute severe onset and others a mild chronic outcome (2, 3). Currently only the draft genome sequence of mastitis-associated E. coli O32:H37 strain P4 has been published (4).

E. coli 1303 was isolated from udder secretions of a cow with clinical mastitis (5) and E. coli ECC-1470 from a chronically infected cow (6). Both genomes were sequenced via whole-genome sequencing with the 454 FLX genome sequencer with GS20 chemistry (Roche Life Science, Mannheim, Germany) to a 27.8-fold or 13.4-fold coverage, respectively. Strain ECC-1470 was also sequenced with a 6-kb insert paired-end (PE) 454 sequencing library. Additionally, Nextera XT chemistry (Illumina, San Diego, CA, USA) for library preparation and a 101-bp PE sequencing run was used to sequence both strains on an Illumina HiScan SQ sequencer.

The 454 reads were *de novo* assembled with Newbler (v2.0.00.20 for 1303 and v2.3 for ECC-1470; Roche). The 454 and Illumina reads were *de novo* assembled using MIRA v3.4.0.1 (7). The hybrid assembly was combined with the initial Newbler assembly within the Gap4 software (v4.11.2) of the Staden package (8). Gaps were closed by primer walking via PCR and Sanger sequencing.

E. coli 1303 possesses a 4,948,797-bp and strain ECC-1470 a 4,803,751-bp chromosome. Each strain harbors an F-plasmid designated p1303_109 (108,501 bp) or pECC-1470_100 (100,061 bp), respectively. Additionally, strain 1303 contains a bacteriophage P1-like plasmid p1303_95 (94,959 bp) and a small cryptic plasmid p1303_5 (4,671 bp).

Annotation was done with Prokka v1.9 (9) and E. coli K-12 MG1655 (NC_000913.3) as reference. Annotations were manually curated by employing the Swiss-Prot, TrEMBL (10), IMG/ER (11), and Ecocyc databases (12). Open reading frame (ORF) finding was verified with YACOP v1 (13) and the reference strain MG1655’s annotation using ACT v12.1.1 (14) for manual curation with Artemis v15.1.1 (15) and tbl2tab v0.1 (https://github.com/aleimba/bac-genomics-scripts/tree/master/tbl2tab). A total of 4,734 coding DNA sequences (CDS) were identified in E. coli 1303 with 22 rRNAs and 91 tRNAs (via tRNAscan-SE v1.3.1 [16]). The E. coli ECC-1470 genome includes 4,506 CDS with 22 rRNAs and 90 tRNAs.

By assigning multilocus sequence types (STs) using ecoli_mlst v0.3 (https://github.com/aleimba/bac-genomics-scripts/tree/master/ecoli_mlst) strains 1303 and ECC-1470 were allocated to phylogroups A (ST10) and B1 (ST847), respectively (17).

The most prominent virulence factors in both strains are the enterobactin siderophore, the group 4-capsule, and the E. coli type III secretion system 2. The genes *flu* (Ag43), *astA* (enteroaggregative E. coli heat-stable enterotoxin 1), *iss* (increased serum survival), an AMR-SSuT genomic island (antimicrobial resistance to streptomycin, sulfonamide, and tetracycline), and the second flagellar cluster, Flag-2, are only present in E. coli 1303. Putative virulence factors that are only present in strain ECC-1470 are two type VI secretion systems, the long polar fimbriae, Pix fimbriae, and the alternative flagellin Flk.

### Nucleotide sequence accession numbers.

The genome sequences have been deposited at DDBJ/ENA/GenBank under the accession numbers CP009166 to CP009169 (strain 1303) and CP010344 and CP010345 (strain ECC-1470).
